# Supplementary material for: Novel Driver Strength Index highlights important cancer genes in TCGA PanCanAtlas patients
Source: PeerJ. 2022 Aug 11;10:e13860. doi: 10.7717/peerj.13860 (PMC9375969; doi:10.7717/peerj.13860)
Supplement: Supplemental Information 2 [file peerj-10-13860-s002.zip › COHORTS/genes plots/cohorts/2021_8_16_14_9_distribution_cohorts_BLCA.pdf]

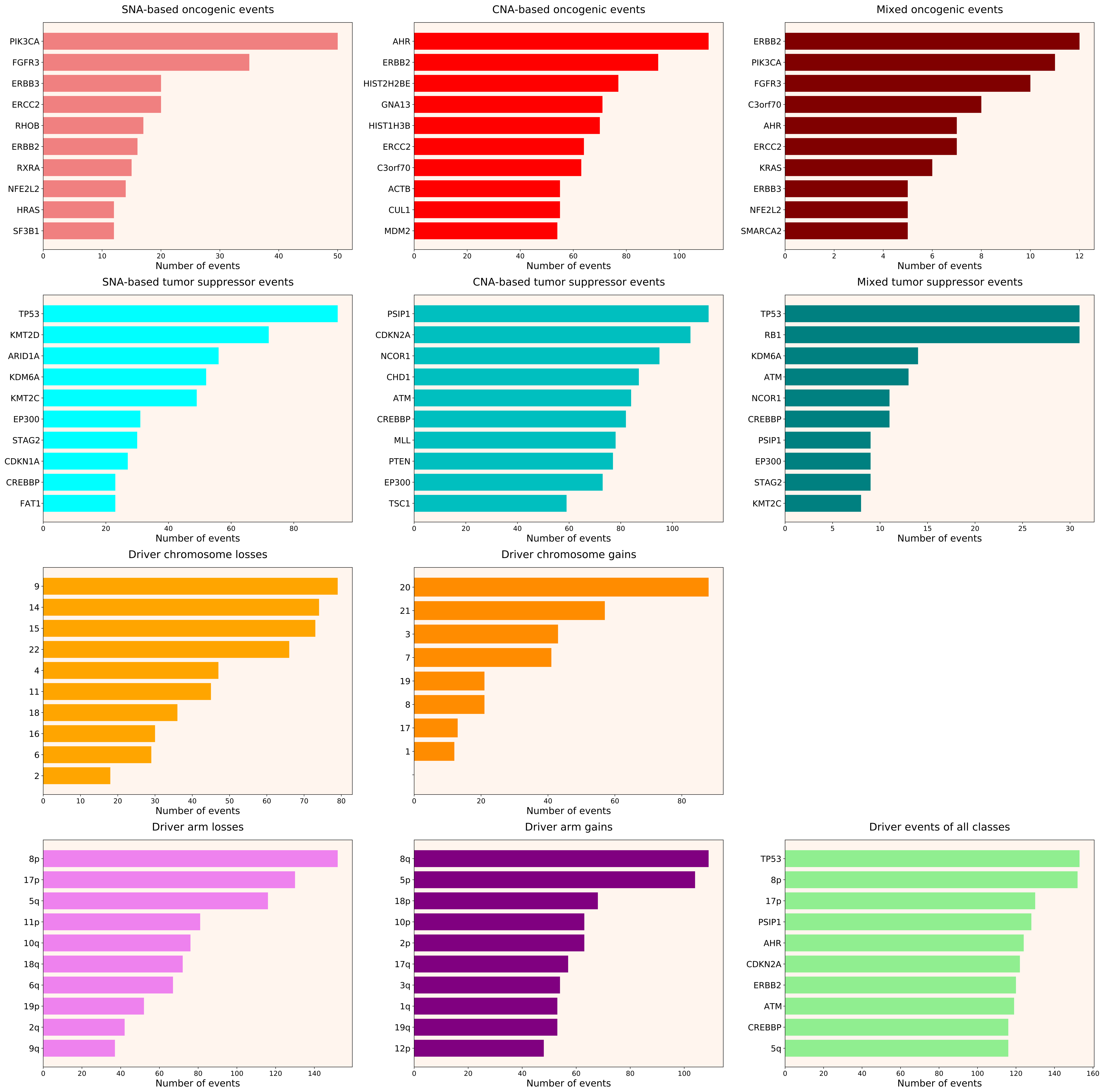

SNA-based oncogenic events
PIK3CA
FGFR3
ERBB3
ERCC2
RHOB
ERBB2
RXRA
NFE2L2
HRAS
SF3B1
0 10 20 30 40 50
Number of events
CNA-based oncogenic events
AHR
ERBB2
HIST2H2BE
GNA13
HIST1H3B
ERCC2
C3orf70
ACTB
CUL1
MDM2
0 20 40 60 80 100
Number of events
Mixed oncogenic events
ERBB2
PIK3CA
FGFR3
C3orf70
AHR
ERCC2
KRAS
ERBB3
NFE2L2
SMARCA2
0 2 4 6 8 10 12
Number of events
SNA-based tumor suppressor events
TP53
KMT2D
ARID1A
KDM6A
KMT2C
EP300
STAG2
CDKN1A
CREBBP
FAT1
0 20 40 60 80
Number of events
CNA-based tumor suppressor events
PSIP1
CDKN2A
NCOR1
CHD1
ATM
CREBBP
MLL
PTEN
EP300
TSC1
0 20 40 60 80 100
Number of events
Mixed tumor suppressor events
TP53
RB1
KDM6A
ATM
NCOR1
CREBBP
PSIP1
EP300
STAG2
KMT2C
0 5 10 15 20 25 30
Number of events
Driver chromosome losses
9
14
15
22
4
11
18
16
6
2
0 10 20 30 40 50 60 70 80
Number of events
Driver chromosome gains
20
21
3
7
19
8
17
1
0 20 40 60 80
Number of events
Driver arm losses
8p
17p
5q
11p
10q
18q
6q
19p
2q
9q
0 20 40 60 80 100 120 140
Number of events
Driver arm gains
8q
5p
18p
10p
2p
17q
3q
1q
19q
12p
0 20 40 60 80 100
Number of events
Driver events of all classes
TP53
8p
17p
PSIP1
AHR
CDKN2A
ERBB2
ATM
CREBBP
5q
0 20 40 60 80 100 120 140 160
Number of events
